# Knowledge, attitudes, and concerns about psilocybin and MDMA as novel therapies among U.S. healthcare professionals

**DOI:** 10.1038/s41598-024-78736-1

**Published:** 2024-11-14

**Authors:** Erin Wang, David S. Mathai, Natalie Gukasyan, Sandeep Nayak, Albert Garcia-Romeu

**Affiliations:** 1 Department of Psychiatry and Behavioral Sciences, Johns Hopkins University School of Medicine, Baltimore, MD USA; 2 Sattva Medicine Psychiatry/Psychotherapy Practice, Miami, FL USA; 3 Ethical Legal Implications of Psychedelics in Society Program, Center for Medical Ethics and Health Policy, Baylor College of Medicine, Houston, TX USA; 4 Department of Psychiatry, Columbia University Medical Center, New York State Psychiatric Institute, New York, NY USA; 5 Center for Psychedelic and Consciousness Research, Johns Hopkins University School of Medicine, Baltimore, MD USA

**Keywords:** Psychedelic, Psilocybin, MDMA, Hallucinogen, Attitudes, Psychology, Therapeutics, Health care, Health policy

## Abstract

Psychedelic-assisted therapy (PAT) with substances like psilocybin and MDMA has shown promise for conditions including depression and post-traumatic stress disorder. Psilocybin and MDMA may become approved medicines in the coming decade. This study assessed knowledge and attitudes regarding PAT among 879 U.S. healthcare professionals via anonymous online survey. Multivariable linear regression was used to identify predictors of openness to clinical use. Most participants (71.2%) were female and White (85.8%), with a mean (SD) age of 45.5 (12.7) years. Registered nurses (25.4%) and physicians (17.7%) comprised the largest professional groups. Respondents endorsed strong belief in therapeutic promise, and moderate openness to clinical use and support for legal access to both substances, with higher overall ratings for psilocybin compared to MDMA. Objective knowledge items revealed low knowledge of therapeutic uses, risks, and pharmacology. Primary concerns were lack of trained providers, financial cost, and potential contraindications. Prior psychedelic use, self-rated knowledge, younger age, and professional role predicted openness to clinical use of psilocybin and MDMA, with physicians reporting lower openness. As psychedelics continue to garner popular and scientific interest, results indicate a pressing need for additional formal training to provide balanced, evidence-based information from trusted sources.

**Supplementary Information:**

The online version contains supplementary material available at 10.1038/s41598-024-78736-1.

## Introduction

Hallucinogens comprise a diverse class of psychoactive drugs that can produce altered states of consciousness involving major changes in thought, mood, and perception via various mechanisms of action^1^. Many indigenous cultures have used plant-based hallucinogens in religious or spiritual ceremonies for centuries^2^. During the 1950s and 60s, there was a wave of scientific research on the classic serotonergic hallucinogens (also known as classic psychedelics) such as psilocybin and lysergic acid diethylamide (LSD), but by the 1970s, research in the United States largely ended due to widespread stigma and increased regulation^3^.

The past few decades have seen a resurgence of clinical research on classic and non-classic hallucinogens (hereafter “psychedelics”), including psilocybin and 3,4-methylenedioxymethamphetamine (MDMA). When administered in controlled settings with psychological support, these substances have shown therapeutic promise for mental health conditions such as depression^4–6^, anxiety^7,8^, post-traumatic stress disorder (PTSD)^9,10^, and substance use disorders (Table 1)^11–13^.

**Table 1:** Summary of current findings on psilocybin and MDMA^14,15^.

Substance	Class	Proposed Mechanism of Action	Subjective Effects	Adverse Effects	Indications Showing Therapeutic Promise
**Psilocybin**	Classic psychedelic	Serotonin 5-HT_2A,_ 5-HT_1A_ and 5-HT_2C_ receptor agonist	Altered perception of time, space, and reality^31,42^; changes in mood and affect; spiritual or mystical experiences;^48,49^enhanced empathy^50^; increased cognitive flexibility^51,52^	Increased blood pressure and heart rate, transient anxiety, panic or paranoia, headache	Depression, anxiety, substance use disorders
**MDMA**	Entactogen^53^	Mixed serotonin, norepinephrine, and dopamine reuptake inhibition and release; serotonin 5-HT_2A_ agonist	Increased sociability and energy;^54^feelings of empathy and connectedness to others^55,56^	Increased blood pressure and heart rate,^51^headache, elevated temperature^56^	PTSD^10,57^

Some benefits of “psychedelic-assisted therapy” (PAT) using substances such as psilocybin and MDMA are thought to derive from the altered states they can elicit that may facilitate increased acceptance and processing of emotions, connectedness to others, forgiveness, self-compassion, cognitive flexibility, insights into the self, and positive changes in worldview^14^. These effects may be further reinforced when combined with psychotherapy, which provides a supportive framework to help individuals navigate the psychedelic experience, integrate insights, and promote lasting positive changes^14–17^. However, this dimension of PAT, which can involve lengthy (i.e., 6–8 h), unpredictable, and emotionally intense drug dosing sessions, may be novel to providers without specialized training.

In light of recent promising clinical trial data, the U.S. Food and Drug Administration (FDA) granted breakthrough therapy designation to psilocybin and MDMA for the treatment of depression and PTSD, respectively^9^. In August 2024, however, the FDA decided not to approve MDMA-assisted therapy for PTSD and requested an additional Phase 3 trial to study the safety and efficacy of the drug^18^. Despite this ruling, scientific and popular interest in PAT has grown significantly in recent years and several studies have been conducted to assess healthcare professionals’ attitudes about the therapeutic potential of these substances. Healthcare providers’ beliefs and attitudes can shape patient care, treatment recommendations, and overall implementation of therapeutic advancements. Furthermore, PAT requires substantial resources and trained personnel, which may include psychiatrists, psychologists, licensed therapists, pharmacists, nurses, integrative medical practitioners, and other healthcare professionals. Therefore, examining healthcare providers’ knowledge base, concerns, and openness to the therapeutic potential of psychedelics is essential for the successful clinical implementation of this new treatment model.

Previous studies have shown that psychiatrists, psychologists, and mental health counselors endorse cautiously favorable attitudes towards psychedelic therapies, indicating they believe psychedelics show treatment promise and support federal funding for medical psychedelic research^19–24^. Participants also expressed concerns about possible psychiatric and neurocognitive risks, lack of trained PAT providers, and the logistics of PAT delivery^20,23^. Available data suggest younger and male individuals were less concerned about the risks of psychedelics and more optimistic about their therapeutic potential^19,22,24^. However, both objective assessment and self-report showed substantial limitations in participants’ evidence-based knowledge on psychedelics^20,23,24^. Greater self-reported knowledge of psychedelics and their use in mental healthcare, as well as personal experience, were correlated with more positive attitudes^21,24^. Studies that surveyed healthcare professionals’ attitudes, knowledge, and beliefs towards specific substances, rather than psychedelics broadly, have demonstrated similar findings^25–31^.

The primary objectives of the present study were to (1) survey a sample of U.S. healthcare professionals to assess their knowledge and attitudes regarding PAT involving psilocybin and MDMA, and (2) to inform future directions for clinical training, policymaking, and medical implementation of PAT based on these data. Ultimately, this research strives to present a more comprehensive picture of the current opportunities and challenges surrounding the clinical use of psychedelics.

## Results

### Participants

Healthcare providers were recruited for this anonymous online survey through the Johns Hopkins Center for Psychedelic and Consciousness Research website and social media advertisements. Of 879 participants, 626 (71.2%) were female and 754 (85.8%) were White (Table 2). Mean (SD) age was 45.5 (12.7) years. Registered nurses comprised the largest professional group (*n* = 223, 25.4%), followed by physicians (*n* = 156, 17.7%). The most represented physician specialties were psychiatry (*n* = 42, 26.9%), family medicine (*n* = 26, 16.7%), and internal medicine (*n* = 25, 16.0%). A total of 291 participants (33.1%) held prescribing capabilities, 807 (91.8%) currently practiced clinically, and 185 (21.0%) conducted research. Previous psychedelic use was reported by 640 participants (72.8%).

**Table 2:** Demographic characteristics.

	*N* = 879
**Age (years)**	
Mean (SD)	45.5 (12.7)
**Gender**	
Female	626 (71.2%)
Male	236 (26.8%)
Non-binary	15 (1.7%)
Prefer not to answer	2 (0.2%)
**Race**	
White	754 (85.8%)
American Indian	3 (0.3%)
Asian Pacific Islander	27 (3.1%)
Black	15 (1.7%)
Multiracial	30 (3.4%)
None of the above	32 (3.6%)
Prefer not to answer	18 (2.0%)
**Hispanic Ethnicity**	67 (7.6%)
**Highest Level of Education**	
Associate degree	52 (5.9%)
Bachelor’s degree	172 (19.6%)
Master’s degree	336 (38.2%)
Doctorate degree	235 (26.7%)
Professional degree	71 (8.1%)
Some college credit, no degree	6 (0.7%)
Trade/technical/vocational training	7 (0.8%)
**Profession**	
Advanced Practice Registered Nurse	23 (2.6%)
Counselor	51 (5.8%
Emergency Medical Technician	12 (1.4%)
Nurse Practitioner	83 (9.4%)
Pharmacist	16 (1.8%)
Physician	156 (17.7%)
Physician Assistant	23 (2.6%)
Psychologist	55 (6.3%)
Registered Nurse	223 (25.4%)
Social Worker	66 (7.5%)
Therapist	86 (9.8%)
Other	85 (9.7%)
**Prescribing Capability**	291 (33.1%)
**Currently Practicing Clinically**	807 (91.8%)
**Conducting Research**	185 (21.0%)
**Ever Taken a Hallucinogen**	650 (72.8%)

A total of 264 participants (30.0%) reported that they have patients that currently use psilocybin, and 160 (18.2%) reported that they have patients that use MDMA. Most participants reported having seen someone under the influence of psilocybin (*n* = 650, 73.9%) and MDMA (*n* = 522, 59.4%) in a recreational context. The majority of these observed experiences were positive for psilocybin (*n* = 617, 89.8%) and MDMA (*n* = 457, 79.6%), but negative experiences were also reported for psilocybin (*n* = 23, 3.3%) and MDMA (*n* = 54, 9.4%).

### Self-reported and objective knowledge

Participants rated how strongly they agreed with statements representing their knowledge and attitudes about the clinical use and legal accessibility of each substance on a 5-point Likert scale ranging from 1 (strongly disagree) to 5 (strongly agree). On average, participants rated their self-reported knowledge as highest regarding psilocybin and MDMA therapeutic indications (3.99 and 3.06, respectively), followed by knowledge on risks (3.65, 3.05), and finally on mechanism of action for each substance (3.08, 2.69) (Table 3).

**Table 3:** Knowledge on psilocybin and MDMA and appropriate settings for clinical administration.

	Psilocybin	MDMA
**Self-Reported Knowledge**^a^ **(Mean (SD))**		
Therapeutic indications	3.99 (0.98)	3.06 (1.27)
Risks and Adverse Effects	3.65 (1.10)	3.05 (1.23)
Mechanism of Action	3.08 (1.13)	2.69 (1.16)
**% Correct Responses on Objective Knowledge Checks, n (%)** ^**b**^		
Therapeutic indications	181 (20.6%)	22 (2.5%)
Risks and Adverse Effects	315 (35.8%)	295 (33.6%)
Mechanism of Action	495 (56.3%)	428 (48.7%)
**Current Knowledge Sources**		
Popular media	611 (69.5%)	543 (61.8%)
Academic literature	577 (65.6%)	421 (47.9%)
Personal experience	516 (58.7%)	332 (37.8%)
Informal conversations	450 (51.2%)	460 (52.3%)
Conferences	232 (26.4%)	204 (23.2%)
Past experience with patients	168 (19.1%)	131 (14.9%)
Colleagues	167 (19.0%)	146 (16.6%)
Formal clinical training	98 (11.1%)	105 (11.9%)
**Trusted Knowledge Sources**		
Experienced clinicians	800 (91.0%)	786 (89.4%)
Academic research centers	792 (90.1%)	814 (92.6%)
Professional organizations	672 (76.5%)	674 (76.7%)
Private training institutions	398 (45.3%)	429 (48.8%)
Pharmaceutical companies	67 (7.6%)	71 (8.1%)
**Appropriate Clinical Administration Settings**		
Specialized clinic	825 (93.9%)	792 (90.1%)
Private practice	691 (78.6%)	606 (68.9%)
Patient’s home (supervised)	662 (75.3%)	574 (65.3%)
Outpatient clinic	558 (63.5%)	514 (58.5%)
Detox facility	538 (61.2%)	456 (51.9%)
Inpatient setting	444 (50.5%)	446 (50.7%)
Home (unsupervised)	185 (21.1%)	113 (12.9%)
Emergency department	64 (7.3%)	59 (6.7%)
None	2 (0.2%)	12 (1.4%)

^a^ Rated on 5-point Likert scale ranging from 1 “strongly disagree” to 5 "strongly agree." ^b^ Based on Objective Knowledge check items (see Supplement A).

Self-reported knowledge ratings were inconsistent with responses on objective knowledge check items. Respondents scored the lowest on their knowledge of the therapeutic indications of psilocybin and MDMA (20.6% and 2.5%, respectively), despite self-rated knowledge being the highest in this domain. Only 5.5% of respondents answered all 3 knowledge check questions correctly for psilocybin, compared to just 1.1% for MDMA. 35% of respondents answered all 3 questions incorrectly for MDMA, compared to 25.5% for psilocybin.

Respondents scored lower on the MDMA questions compared to psilocybin, consistent with self-rated knowledge. Self-rated and objective knowledge scores were significantly, but weakly correlated (0.26; 95% CI: 0.19, 0.32, *p* < 0.001 for psilocybin and 0.31; 95% CI: 0.25, 0.37, *p* < 0.001 for MDMA).

Mean self-rated knowledge of psilocybin was higher on average for physicians compared to other professionals, though this did not reach statistical significance (*p* = 0.13). Mean self-rated knowledge of MDMA was higher for physicians compared to advanced practice providers (APPs) (*p* = 0.02), nurses (*p* < 0.001), and mental health professionals (*p* = 0.02). Compared to other professionals, a greater proportion of physicians responded correctly to at least 2 out of 3 knowledge check questions for both psilocybin and MDMA. This aligns with the higher self-rated knowledge endorsed by physicians relative to other professionals. All professions scored lower on the MDMA knowledge check questions compared to the psilocybin questions.

### Primary and trusted sources of knowledge

Popular media (69.5% for psilocybin, 61.8% for MDMA) and academic literature (65.6%, 47.9%) were respondents’ main current sources of knowledge, followed by informal conversations (51.2%, 52.3%) and personal experience (58.7%, 37.8%). Reported sources of knowledge were overall similar for psilocybin and MDMA (Table 3). More respondents learned about psilocybin through academic literature and personal experience, compared to MDMA.

The most trusted sources of knowledge for psilocybin and MDMA were academic research centers (91.0% for psilocybin, 89.4% for MDMA), experienced clinicians or practitioners (90.1%, 92.6%), and professional organizations (76.5%, 76.7%). Only 7.6% and 8.1% of respondents stated they would trust pharmaceutical companies to provide information on psilocybin and MDMA. Responses were overall similar for psilocybin and MDMA.

### Attitudes regarding psilocybin and MDMA

Overall internal consistency was high within grouped knowledge and attitude domains, with Cronbach’s α ranging from 0.73 to 0.91, except for openness to clinical use of psilocybin (0.69) and support for legal access to MDMA (0.51) (Supplemental Table S1).

**Fig. 1:**
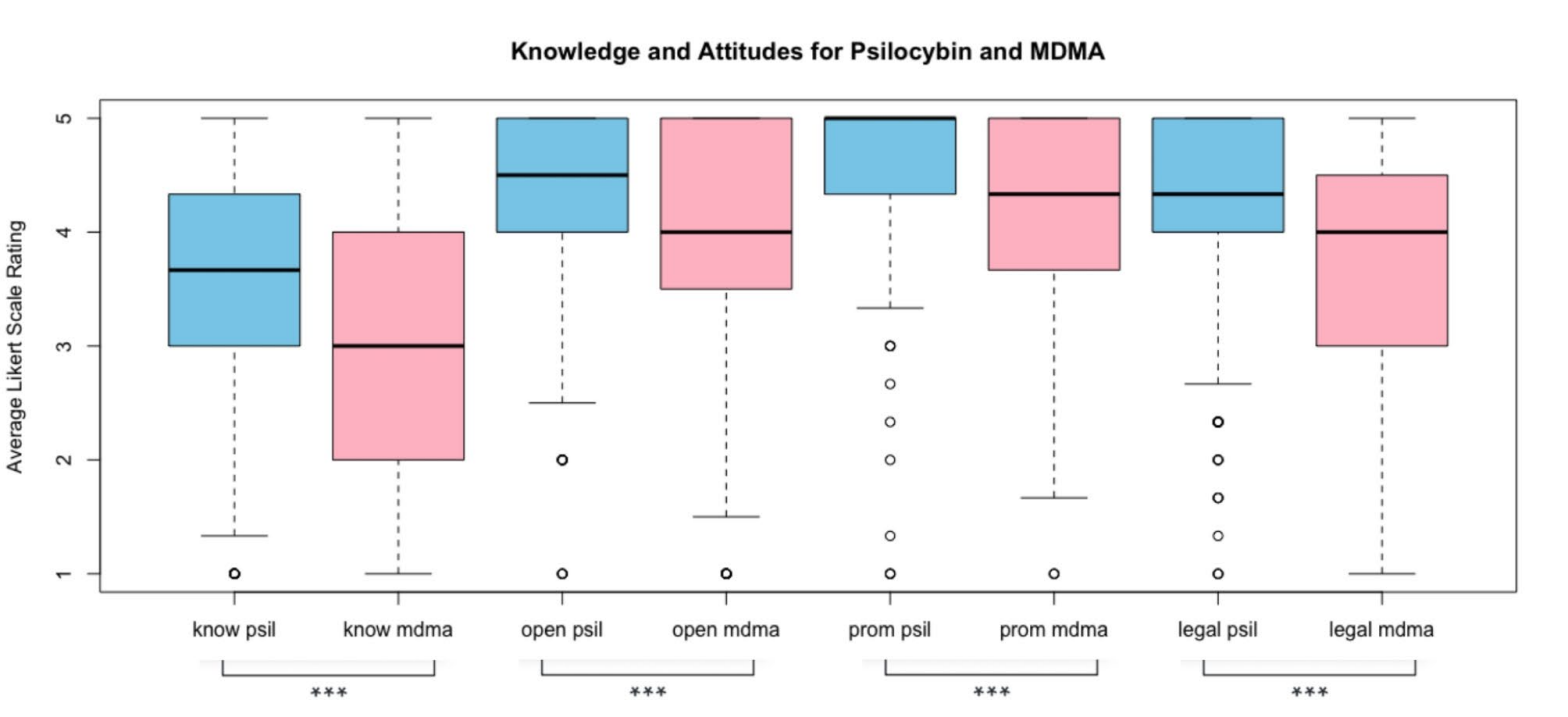
Knowledge and attitude ratings for psilocybin and MDMA. Average self-rated knowledge (know), openness to clinical use (open), belief in therapeutic promise (prom), and support for legal access (legal) to psilocybin and MDMA are shown. A 5-point Likert scale rating was used (1 = strongly disagree and 5 = strongly agree). Paired t-tests showed that the average rating was significantly higher for psilocybin compared to MDMA across all 4 categories (p-value < 0.001). ***p-value < 0.001 for paired t-test.

*Therapeutic Promise.* Belief in therapeutic promise of psilocybin and MDMA was high across all professions (Table 4). Overall, mean (SD) ratings for belief in therapeutic promise were 4.67 [0.53] for psilocybin, and 4.25 [0.73] for MDMA, indicating an average response between “Agree” and “Strongly Agree.” Specifically, 93% believed psilocybin can be delivered safely in clinical settings and 76% felt MDMA could be delivered safely in clinical settings. Similarly, 95% endorsed the therapeutic promise of psilocybin, with 73% endorsing therapeutic benefits of MDMA. Finally, 98% supported further psilocybin research and 92% called for more research on MDMA. Belief in therapeutic promise of psilocybin was significantly higher than for MDMA (*p* < 0.001, Fig. 1).

**Table 4:** Knowledge, attitudes, and concerns regarding psilocybin and MDMA by Profession.

	Psilocybin (mean, SD)^a^	MDMA (mean, SD)^a^
**Self-rated Knowledge**	3.57 (0.94)	2.93 (1.12)
Physicians	3.73 (1.04)	3.27 (1.18)
Advanced practice providers^b^	3.58 (0.93)	2.86 (1.15)
Registered nurses	3.50 (0.87)	2.75 (1.09)
Mental health professionals^b^	3.52 (0.91)	2.92 (1.05)
Other^b^	3.63 (1.00)	2.95 (1.15)
**Openness to Clinical Use**	**4.47 (0.69)**	**3.98 (0.92)**
Physicians	4.39 (0.74)	3.88 (1.05)
Advanced practice providers	4.52 (0.68)	4.00 (0.94)
Registered nurses	4.52 (0.61)	4.00 (0.77)
Mental health professionals	4.51 (0.66)	4.06 (0.94)
Other	4.31 (0.79)	3.84 (0.91)
**Belief in Therapeutic Promise**	**4.67 (0.53)**	**4.25 (0.73)**
Physicians	4.63 (0.57)	4.22 (0.79)
Advanced practice providers	4.58 (0.60)	4.14 (0.84)
Registered nurses	4.72 (0.47)	4.21 (0.65)
Mental health professionals	4.69 (0.51)	4.34 (0.70)
Other	4.69 (0.56)	4.26 (0.67)
**Support for Legal Access**	**4.30 (0.71)**	**3.74 (0.80)**
Physicians	4.12 (0.78)	3.61 (0.80)
Advanced practice providers	4.15 (0.78)	3.59 (0.85)
Registered nurses	4.41 (0.62)	3.72 (0.75)
Mental health professionals	4.32 (0.68)	3.84 (0.79)
Other	4.47 (0.71)	3.88 (0.83)
**Concerns**		
***Lack of trained providers***		
Physicians	3.71 (1.00)	3.64 (1.06)
Advanced practice providers	3.56 (1.05)	3.73 (1.05)
Registered nurses	3.43 (1.11)	3.56 (1.08)
Mental health professionals	3.86 (0.97)	3.89 (1.01)
Other	3.47 (1.20)	3.48 (1.23)
***Financial cost / insurance coverage***		
Physicians	2.85 (1.32)	2.85 (1.25)
Advanced practice providers	3.24 (1.16)	3.19 (1.18)
Registered nurses	2.97 (1.28)	2.93 (1.17)
Mental health professionals	3.21 (1.22)	3.24 (1.18)
Other	2.88 (1.29)	2.91 (1.26)
***Administration to patients with contraindications***		
Physicians	3.03 (1.08)	3.13 (1.16)
Advanced practice providers	3.15 (1.13)	3.21 (1.01)
Registered nurses	2.96 (1.09)	3.03 (1.13)
Mental health professionals	3.37 (0.98)	3.34 (1.07)
Other	3.02 (1.17)	3.12 (1.18)
***Exploitation of patients***		
Physicians	3.05 (1.29)	3.12 (1.29)
Advanced practice providers	2.85 (1.23)	3.09 (1.19)
Registered nurses	2.69 (1.33)	2.84 (1.30)
Mental health professionals	3.00 (1.28)	3.19 (1.32)
Other	2.72 (1.32)	2.89 (1.33)
***Psychosis***		
Physicians	2.83 (1.15)	2.64 (1.27)
Advanced practice providers	2.81 (1.07)	2.87 (1.16)
Registered nurses	2.74 (1.09)	2.59 (1.17)
Mental health professionals	3.10 (1.14)	2.95 (1.24)
Other	2.57 (1.02)	2.55 (1.19)
***Time required to administer***		
Physicians	2.61 (1.12)	2.39 (1.06)
Advanced practice providers	2.49 (1.07)	2.57 (1.16)
Registered nurses	2.08 (1.07)	2.13 (1.07)
Mental health professionals	2.37 (1.13)	2.42 (1.16)
Other	2.37 (1.14)	2.26 (1.09)
***Stigma***		
Physicians	2.18 (1.06)	2.31 (1.20)
Advanced practice providers	2.20 (1.13)	2.52 (1.28)
Registered nurses	2.19 (1.17)	2.35 (1.23)
Mental health professionals	2.09 (1.05)	2.21 (1.08)
Other	2.46 (1.28)	2.47 (1.28)
***Recreational use / misuse***		
Physicians	2.38 (1.00)	3.10 (1.12)
Advanced practice providers	2.47 (1.16)	3.02 (1.11)
Registered nurses	2.12 (1.00)	2.88 (1.07)
Mental health professionals	2.31 (1.04)	2.93 (1.10)
Other	2.23 (1.09)	2.88 (1.13)
***Addiction***		
Physicians	1.45 (0.82)	2.36 (1.12)
Advanced practice providers	1.63 (1.02)	2.49 (1.12)
Registered nurses	1.41 (0.75)	2.28 (1.11)
Mental health professionals	1.57 (0.95)	2.33 (1.09)
Other	1.56 (0.88)	2.30 (1.08)

^a^ Rated on 5-point Likert scale ranging from 1 “strongly disagree” to 5 "strongly agree."^b^ Professions were grouped as such: Advanced Practice Provider = Physician Assistant, Nurse Practitioner, Advanced Practice Registered Nurse. Mental Health Professional = Psychologist, Counselor, Therapist, Social Worker. Other = all others (e.g. EMT, pharmacist).

*Openness to Clinical Use.* Openness to clinical use was rated a mean of 4.47 [0.69] for psilocybin and 3.98 [0.92] for MDMA. A majority endorsed openness to using psilocybin (89%) and MDMA (67%), and a majority also expressed an interest in further training to use psilocybin (90%) and MDMA (79%) in their practice. Openness to clinical use of psilocybin was lower among physicians compared to other professions. Openness to clinical use of psilocybin was significantly higher than for MDMA (*p* < 0.001, Fig. 1).

*Support for Legal Access.* 96% of participants supported legal medical use of psilocybin and 84% supported legal medical use of MDMA. Recreational non-medical use had less support with 61% feeling psilocybin should be legally accessible for recreational/non-medical use compared to 36% endorsing the same for MDMA. Finally, 84% noted support for legal access to psilocybin for religious use (not queried regarding MDMA). Mean support for legal access to psilocybin and MDMA (i.e., across all settings) was higher for registered nurses (RNs), mental health professionals (MHPs), and other professionals than for physicians and advanced practice providers (APPs). Support for legal access to psilocybin was significantly greater than for MDMA (*p* < 0.001, Fig. 1).

**Fig. 2:**
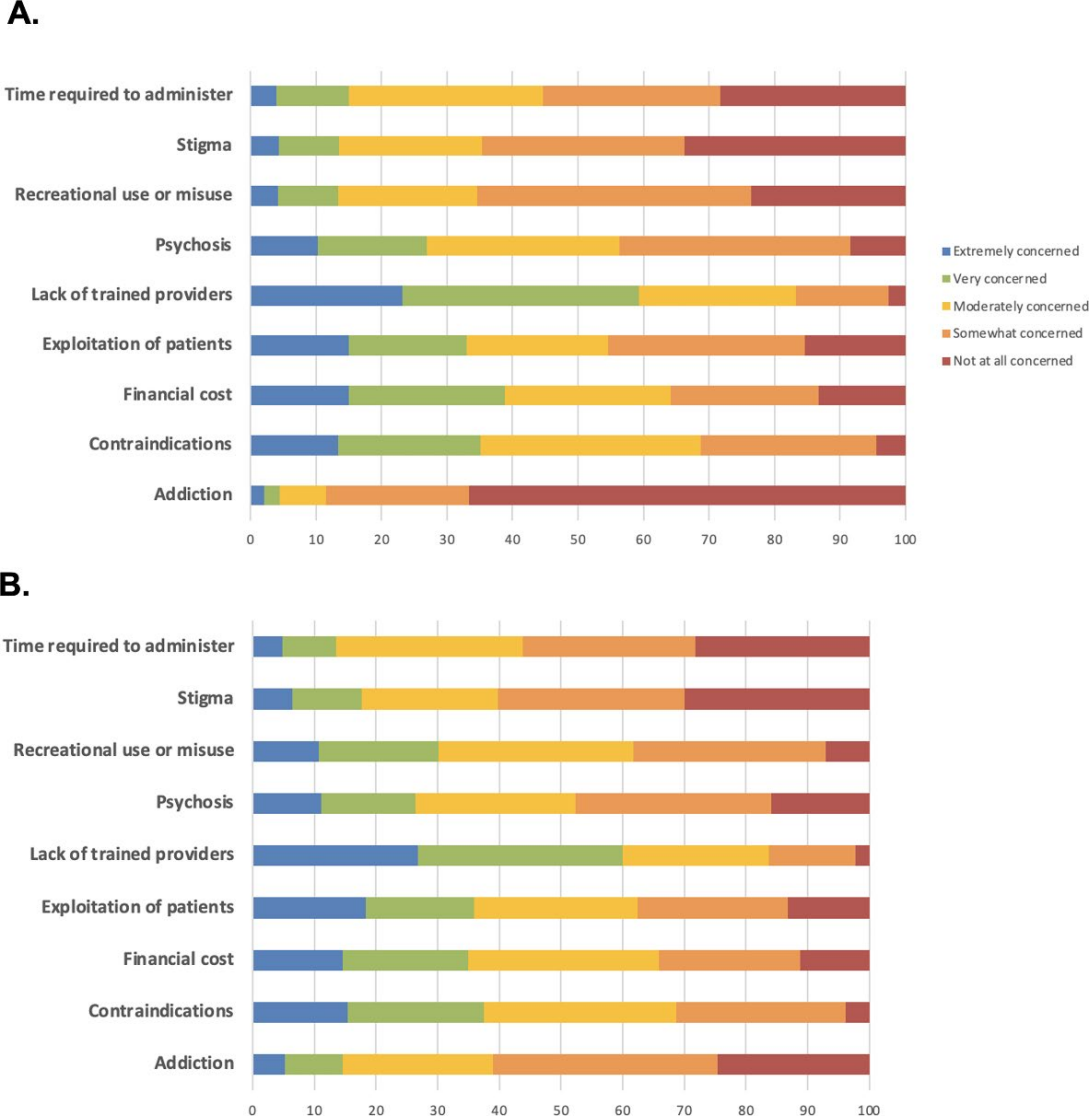
Concern ratings for psilocybin (**A**) and MDMA (**B**). These figures show the proportion of respondents stating their level of concern (1 = not at all concerned and 5 = extremely concerned) for each of the following potential issues related to psilocybin and MDMA-assisted therapy.

*Concerns about Clinical Use of Psilocybin and MDMA.* The most highly endorsed concerns regarding clinical administration of psilocybin were lack of trained providers (59% responded “extremely concerned” or “very concerned”), followed by financial costs / insurance coverage of psilocybin treatment (39%), potential harms to patients with contraindications (35%), potential exploitation of patients (33%), and potential for inducing psychosis (27%). The amount of time needed for psilocybin treatment (15%), stigma surrounding psilocybin (14%), recreational use of psilocybin (13%), and addictive potential of psilocybin (4%) were rated as less concerning (Fig. 2A).

The most highly endorsed concerns regarding clinical administration of MDMA were lack of trained providers (60%), followed by potential harms to patients with contraindications (38%), potential exploitation of patients (36%), financial costs / insurance coverage of MDMA treatment (35%), and recreational use of MDMA (30%). Potential for inducing psychosis (26%), stigma surrounding MDMA (18%), addictive potential of MDMA (15%), and amount of time needed for MDMA treatment (14%) were rated as less concerning (Fig. 2B).

MHPs reported the highest concern scores on average, followed by physicians and APPs, then RNs (Table 4). MHPs had greater concerns about administration to patients with contraindications, financial costs, and lack of trained providers. Physicians had greater concerns about recreational use and misuse.

### Appropriate settings for psilocybin and MDMA therapies

Respondents regarded specialized clinics (93.9% for psilocybin, 90.1% for MDMA) to be the most appropriate clinical setting for the administration of psilocybin and MDMA, followed by private practice (78.6%, 68.9%), patient’s home with supervision (75.3%, 65.3%), outpatient clinics (63.5%, 58.5%), and detox/drug rehabilitation facilities (61.2%, 51.9%). Responses were similar between psilocybin and MDMA (Table 4). Slightly more respondents favored detox facilities and patient’s home (supervised and unsupervised) for psilocybin compared to MDMA.

#### Predictive models

Multivariable linear regression models were used to determine how professional role, demographic characteristics, knowledge, and personal experience predict healthcare professionals’ openness to clinical use of psilocybin and MDMA. Demographic variables predicted only a small proportion of total variance (R^2^ = 0.172; Supplemental Table S2). For psilocybin, openness to clinical use was significantly associated with prior personal experience using psychedelics and self-rated knowledge of psilocybin. Openness to clinical use of psilocybin significantly decreased as respondent age increased, with respondents who were 18–29 years old being most open to using psilocybin clinically, followed by those in the 30–49, 50–69, and 70 + year old age ranges. Average ratings of concern about psilocybin were not associated with openness to clinical use after adjusting for other covariates. APPs, RNs, and MHPs were more open to clinical use of psilocybin compared to physicians. Sex, race, and level of education were not significant predictors of openness to using psilocybin clinically.

Model findings for MDMA were similar to those of psilocybin. Demographic variables predicted only a small proportion of total variance (R^2^ = 0.204; Supplemental Table S3). For MDMA, openness to clinical use was significantly associated with prior personal experience using psychedelics and self-rated knowledge of MDMA. Openness to clinical use of psilocybin significantly decreased as respondent age increased, with respondents who were 18–29 years old being most open to using MDMA clinically, followed by those in the 30–49, and 50–69 year old age ranges. Average ratings of concern about MDMA were not associated with openness to clinical use after adjusting for other covariates. APPs, RNs, and MHPs were more open to clinical use of MDMA compared to physicians. American Indian race was associated with lower openness to clinical MDMA use. Otherwise, sex, race, and education level were not significant predictors of openness to clinical use of MDMA.

## Discussion

The present study presents findings on healthcare professionals’ knowledge, attitudes, and concerns regarding clinical use of psilocybin and MDMA. Overall, respondents endorsed strong belief in therapeutic promise, moderate openness to clinical use, and support for legal medical access to both of these substances. Ratings of knowledge, openness, and belief in therapeutic promise were higher for psilocybin compared to MDMA for all domains, indicating less familiarity, and greater potential stigma or perceived risk of harm of MDMA as compared to psilocybin. This is noteworthy in light of the recent FDA decision not to approve MDMA-assisted therapy for PTSD, raising the question of whether such relatively negative views surrounding MDMA may have influenced the decision-making process. A key finding highlighted notable discrepancies between self-reported knowledge and performance on objective knowledge checks among the current sample. Despite high ratings in self-reported knowledge, objective knowledge items revealed relatively limited knowledge of the potential therapeutic uses, risks and side effects, and pharmacology of both psilocybin and MDMA, with the majority of respondents answering no more than 1 out of the 3 questions correctly. These data suggest a lack of formalized education in current clinical training programs and highlight potential misinformation disseminated in popular media. Physicians on average scored higher on objective knowledge items and also reported greater concerns about potential recreational use of psilocybin and MDMA than other professions, potentially indicating more comprehensive training and possibly more conservative attitudes than other healthcare professionals.

Notably, popular media and academic literature were the main current sources of knowledge on psilocybin and MDMA cited by participants, followed by informal conversations and personal experience. This raises concerns that medical professionals (and likely the public at large) are primarily getting information about psychedelics from popular media sources that may contain sensationalism, unsubstantiated claims, and even blatant misinformation. Other studies have demonstrated that individuals with low knowledge reported relying on heuristics to inform their opinions, often gaining knowledge from media or news articles^32^. Personal experience did serve as an important form of firsthand experiential knowledge, with 73% of respondents reporting prior psychedelic use (not limited to psilocybin or MDMA), and the majority of respondents having observed someone under the influence of psilocybin and MDMA, largely in recreational settings and in experiences typically described as positive. However, overreliance on potentially biased or ill-informed sources such as popular media and informal conversations among highly trained healthcare professionals also underscores the necessity for more systematic academic and clinical training regarding psychedelics in current curricula.

The most trusted sources of knowledge for psilocybin and MDMA were academic research centers, experienced clinicians or practitioners, and professional organizations, suggesting that these groups should play a key role in providing a solid evidence base on psychedelics, disseminating knowledge, and training future practitioners. Academic institutions could play an especially vital role in developing curricula for healthcare professionals and trainees along with continuing medical education programs to address these educational gaps. Institutions such as Johns Hopkins, Yale, Columbia, and others are currently developing formal training programs to support rigorous education for healthcare professionals and the lay public regarding psychedelics and psychedelic therapies^33–35^. Additionally, professional organizations including the American Psychological Association and American Psychiatric Association are actively working to offer evidence-based educational seminars online and at annual meetings, as well as forthcoming textbooks regarding psychedelic therapy^36,37^. Conversely, pharmaceutical companies were rated lowest in terms of trusted sources of information (7.6% and 8.1% for psilocybin and MDMA, respectively), demonstrating widespread skepticism of for-profit organizations with financial interests in these potential therapeutics.

Major sources of concern for both substances were the lack of trained providers, financial cost and insurance coverage, and administration to patients with contraindications, as well as potential exploitation of patients, which all represent significant issues that must be considered carefully as these interventions gain more traction. Administration to patients with contraindications was a leading concern for MHPs. These professionals are specially trained in mental health, and may be more concerned about the psychiatric risks involved for individuals using psychedelics. Personal or family history of a primary psychotic disorder or bipolar disorder have generally been exclusionary for psilocybin and MDMA clinical trials due to increased risk for rare but serious adverse events such as psychosis and mania^38,39^. Clinical trials have therefore aimed to minimize the risks of PAT by conducting thorough screening prior to enrollment, ensuring adequate training of staff, closely monitoring psychiatric status throughout the duration of study participation, and referring to additional treatment post-trial when needed^17,40^. However, it is unclear how these drugs would be used if they were medically available outside research settings and what risk profiles they may exhibit with more widespread clinical use. As more clinical studies have been conducted, other potential risks have emerged, such as suicidality. If approved as new medicines, such risks would presumably be managed via a Risk Evaluation and Mitigation Strategy (REMS) like that used for FDA-approved esketamine therapy for treatment-resistant depression. Under the current REMS, esketamine is administered at specially approved sites where patients must stay for at least 2 h post-dosing under medical supervision, vital signs are monitored periodically throughout that time, and patient mental status is assessed for adverse effects such as sedation and dissociation to ensure safety before discharge. It is likely that similar provisions would be mandated for federally approved use of psilocybin or MDMA. Additionally, many clinics have evolved to offer ketamine for a range of off-label mental health indications, though it remains to be seen whether such off-label use would be viable for psilocybin or MDMA, and if so, under what safeguards^41^.

Interestingly, two thirds of participants reported no concern about addictive potential of psilocybin, in line with the relatively low risk of dependence and abuse potential of classic psychedelics^42^. Nevertheless, respondents indicated that specialized clinics were the most appropriate clinical setting for the administration of psilocybin and MDMA, followed by private practice clinics, with some feeling these substances could be safely administered at a patient’s home with supervision, outpatient clinics, and detox/drug rehabilitation facilities with appropriate infrastructure and support. These data suggest potential for implementation using a variety of treatment models that could help decrease costs, such as home use with oversight or outpatient group therapy as has been explored with ketamine^43,44^. Several factors were associated with openness to clinical use of psilocybin and MDMA, including prior psychedelic use, self-rated knowledge, younger age, and professional role, with APPs, RNs, and MHPs reporting more openness than physicians, after adjusting for covariates.

Findings from the present study should be considered in light of a number of noteworthy limitations. First, responses may reflect participants’ experiences and observations regarding psilocybin-containing mushrooms rather than synthetic psilocybin used in most clinical trials, which might have inadvertently influenced the study results. Therefore, these findings should be interpreted with caution when considering attitudes towards psilocybin’s potential medical use. Additionally, the study sample was not representative of the general population of healthcare professionals, and instead relied on an anonymous convenience sample of self-reported U.S. healthcare professionals gathered via word of mouth and online advertisements through the Johns Hopkins Center for Psychedelic and Consciousness Research (CPCR) website and social media accounts. This, along with the high reported rate of personal experience with psychedelics, suggests a possible self-selection bias that may have skewed results toward more positive views of the drugs queried. Additionally, CPCR has historically focused on psilocybin research, which could have further biased results in favor of more positive attitudes towards psilocybin over MDMA.

The study sample was also relatively homogeneous, and predominantly comprised of white, female respondents. As such, these findings may present an overly optimistic picture of attitudes towards psychedelics that may vary across providers of different racial and ethnic backgrounds, and those without prior history of psychedelic use. Non-Hispanic Whites have historically been over-represented in psychedelic research, with a 2024 review of 39 psychedelic clinical trials finding that this group comprised a disproportionate majority (85.0%) of study participants^45^. Therefore, the present data fail to adequately capture how ethnoracial minoritized populations and/or individuals without prior psychedelic experience might view these therapies differently. For instance, our finding that American Indian race among the limited sample of respondents here was associated with lower openness to clinical MDMA use suggests the importance of exploring differing cultural perspectives around psychedelics in more depth. Future research studies could specifically sample healthcare professionals from underrepresented ethnoracial groups and those with limited prior experience or knowledge of psychedelics to increase the results’ generalizability and applicability^46,47^. Research institutions should also enhance efforts to build trust and promote involvement among marginalized communities to advance racial equity and diversity in psychedelic research. This will be increasingly important as the field continues to evolve and these substances move toward potential approval as new medicines in the coming decade.

In conclusion, this study adds to the growing body of evidence on current healthcare providers’ views and concerns regarding adoption of novel psychedelic-assisted therapies in clinical practice. The data suggest overarching belief among respondents in the therapeutic promise of psilocybin and MDMA in line with recent clinical trials findings, and openness to using these substances in clinical settings among currently practicing healthcare professionals, with slightly greater openness towards psilocybin overall for reasons that are not entirely clear but warrant further investigation. Notably, data indicate a perceived need and widespread desire for clinical training to use these substances medically. Despite high ratings of self-perceived knowledge of the drugs queried, respondents demonstrated relatively poor understanding in a self-assessment quiz, which further highlights the need for more comprehensive training across disciplines. Concerns were largely centered around lack of trained providers, financial costs and insurance coverage, and potential for patient harm due to malpractice more so than any risks associated with the drugs themselves. Despite advanced training and access to specialized knowledge, healthcare providers most commonly reported popular media as a source of information on psychedelics, rather than professional organizations or scholarly literature. As psychedelics continue to maintain popular and scientific interest, it will be critical to curtail misinformation and sensationalized media while providing balanced, empirically validated information to both healthcare providers and the general public to mitigate public health risks and avoid unrealistic expectations regarding psychedelic-assisted therapies. Academic medical centers and professional organizations, which appear to garner high rates of trust among providers, should therefore work towards developing evidence-based curricula to keep current providers up to date and consider potential training programs for a nascent workforce of practitioners who can safely deliver psychedelic-assisted therapies.

## Methods

### Participants

Our final sample consisted of 879 healthcare providers recruited through the Johns Hopkins Center for Psychedelic and Consciousness Research website, online advertisements, and social media. Online recruitment advertisements sought out healthcare professionals to share their thoughts on medical use of psychedelics (e.g., “Are you a healthcare professional? What are your opinions on medical use of psychedelics?”). In the present study, eligible individuals were: (1) at least 18 years old, (2) could read and write English fluently, and (3) reported working in a clinical setting in the United States as a healthcare professional or mental health provider.

### Procedures

Interested parties were directed to a webpage with study inclusion and exclusion criteria and general information about study participation. Participants completed an anonymous online survey hosted on the secure web-based platform Qualtrics (Qualtrics, 2014). They were informed that this study was deemed exempt by a Johns Hopkins University School of Medicine Institutional Review Board (IRB) as it involves minimal risk and does not directly involve human subjects because no personally identifiable information was collected. All participants were informed of the voluntary nature of the study before proceeding and consented to participate by completing the survey. All research was performed in accordance with relevant guidelines/regulations and in accordance with the Declaration of Helsinki. The data presented here are part of a larger study characterizing knowledge and attitudes about various potential novel therapies including psilocybin, MDMA, ketamine, and cannabis. Data on ketamine and cannabis will be presented in separate manuscripts.

Quality control measures were implemented throughout the survey. Survey answers were rejected if participants: (1) failed to pass an automated public Turing test (“CAPTCHA”) item, (2) answered any of the three attention check items incorrectly, (3) completed the survey more than once, (4) indicated that they had issues completing the survey that would make their responses inaccurate or invalid, (5) indicated that they had trouble understanding the questions in a way that would make their responses inaccurate or invalid, (6) indicated that they did not answer honestly or to the best of their knowledge, or (7) did not complete the entire survey. Only respondents that met all these criteria were included in the analyses presented here.

Data were collected from December 15, 2021 to October 9, 2023. Of 2,212 total responses collected, 1,333 failed to meet established study criteria, leaving a final analysis sample of 879 (40% of the original sample).

### Survey design

The survey was divided into six sections (see Supplement A for the full survey instrument). The first section of the survey was composed of basic demographic questions (i.e. respondents’ age, gender, highest level of education, race, ethnicity, religious affiliation, household income, professional role, specialty (for physicians), year of training completion, geographic region, and whether they have prescribing capabilities, currently practice clinically, conduct research, or have ever taken a hallucinogen).

The next four sections were structured in parallel, with each section containing questions about a particular substance: psilocybin, MDMA, ketamine, and cannabis. Each of these sections was divided into (1) an assessment of psychedelic-related beliefs, attitudes, and experiences, and (2) an objective knowledge check.

In part (1), participants were asked to rate how strongly they agreed with statements representing their knowledge and attitudes about the clinical use and legal accessibility of each substance on a 5-point Likert scale ranging from “strongly disagree” to “strongly agree.” Participants indicated where most of their knowledge on a particular substance came from, which sources they would trust for information on the therapeutic use of that substance, and in what settings it would be appropriate to administer that substance clinically by selecting all options that apply from a checklist. The importance of potential concerns was collected on a 5-point Likert scale ranging from “not at all concerned” to “extremely concerned.” A free text box was provided for participants to share any additional concerns. Respondents were asked if they have ever seen someone under the influence of the substance, with four possible responses (“no, never”; “yes, while they were seeking medical care”; “yes, in a recreational context”; or “yes, in a research/clinical setting”). If participants responded yes to the previous question, they were asked to describe the experience they observed on a 5-point scale ranging from “primarily positive” to “primarily negative.”

In the part (2) objective knowledge check, respondents were asked to select all correct answers from a list of responses when asked about the evidence-based clinical indications, risks, and primary mechanism of action of each substance. A final free text box was provided for participants to share any additional thoughts on the therapeutic use of that substance. Before moving on to the next section, participants were provided peer-reviewed information and resources on the substance queried in that section.

The last section of the survey was a 3-item quality check. Participants were asked if they had issues completing the survey that would make their responses inaccurate or invalid, if they had trouble understanding the questions in a way that would make their responses inaccurate or invalid, or if they did not answer the questions honestly or to the best of their knowledge. Finally, three attention check questions were interspersed throughout the entire survey to further assess the validity of responses.

### Variables

Knowledge and attitude Likert scale responses were grouped into 4 categories representing self-rated knowledge, openness to clinical use, belief in therapeutic promise, and support for legal access (see Supplement B for complete list). Internal consistency (Cronbach’s alpha) was calculated for Likert scale items measuring similar domains (knowledge, openness, belief in therapeutic potential, and legal access). Knowledge check items were scored in the following manner: Selecting the correct answer for each checkbox response (yes or no) earned one point, and the total number of points was summed and divided by the total number of points to determine a percent correct score. Healthcare profession was categorized as: Physician, Advanced Practice Provider (Physician Assistant, Nurse Practitioner, Advanced Practice Registered Nurse), Registered Nurse, Mental Health Professional (Psychologist, Counselor, Therapist, Social Worker), and Other (e.g. EMT, Pharmacist).

### Statistical analysis

Participant demographic characteristics were summarized. We performed descriptive analyses of healthcare providers’ attitudes, concerns, and knowledge about psilocybin and MDMA. Responses to questions about personal and professional exposure, knowledge and attitude ratings, concern ratings, knowledge check scores, sources of knowledge, trusted sources of knowledge, and appropriate clinical administration settings were tabulated. Pearson correlation of self-rated knowledge and objective knowledge check scores were computed for psilocybin and MDMA. Paired t-tests were performed to compare total scores on the 4 knowledge and attitude domains between psilocybin and MDMA. Knowledge and attitude ratings, concern ratings, and knowledge check scores by professional role were also calculated.

Finally, multivariable linear regression was performed to conduct a predictive analysis of healthcare providers’ openness to clinical use of psilocybin and MDMA based on their demographic characteristics, personal experience, self-rated knowledge, concern ratings, and profession. Two separate models were run for psilocybin and MDMA. The outcomes of interest were (1) openness to clinical use of psilocybin and (2) openness to clinical use of MDMA. Covariates included age group, sex, race, education level, profession, personal experience using psychedelics, self-rated knowledge, and average self-rated concern.

## Electronic supplementary material

Below is the link to the electronic supplementary material.

Supplementary Material 1

Supplementary Material 2

## Data Availability

The datasets generated and analyzed during the current study are available from the corresponding authors on reasonable request.
