# Supplementary material for: Knowledge, attitudes, and concerns about psilocybin and MDMA as novel therapies among U.S. healthcare professionals
Source: Sci Rep. 2024 Nov 14;14:28022. doi: 10.1038/s41598-024-78736-1 (PMC11564663; doi:10.1038/s41598-024-78736-1)

**Supplemental Materials**

**Supplement A: Survey Instrument (uploaded as separate document)**

**Supplement B: Grouped Knowledge and Attitude Likert Scale Questions**

Self-rated knowledge:

1. I have good knowledge of the potential therapeutic uses of psilocybin.
2. I have good knowledge of the risks and side effects of psilocybin.
3. I have good knowledge of the pharmacology of psilocybin.

Openness to clinical use:

1. I would be open to using psilocybin clinically.
2. I would like to receive training on using psilocybin as a treatment with my patients.

Belief in therapeutic promise:

1. Psilocybin can be delivered safely in a clinical setting.
2. Psilocybin shows promise in treating psychiatric disorders.
3. Psilocybin deserves further research for treatment of psychiatric disorders

Support for legal access:

1. Psilocybin should be legally accessible for spiritual/religious use. (not asked for MDMA)
2. Psilocybin should be legally accessible for recreational/non-medical use.
3. Psilocybin should be legally accessible for supervised medical use.

**Table S1. Internal Consistency Across Domains (Cronbach's α)**

|  | **Psilocybin** | **MDMA** |
| --- | --- | --- |
| Self-rated knowledge | 0.85 | 0.91 |
| Openness to clinical use | 0.69 | 0.83 |
| Belief in therapeutic promise | 0.83 | 0.87 |
| Support for legal access | 0.73 | 0.51 |

**Table S2. Psilocybin multivariable linear regression model results.**

| **Coefficient** | **Estimate** | **Standard Error** | **t value** | **Pr(>\|t\|)** | **Significance** |
| --- | --- | --- | --- | --- | --- |
| Intercept | 3.509 | 0.157 | 22.329 | <2e-16 | *** |
| Profession: APP | 0.164 | 0.075 | 2.182 | 0.029 | * |
| Profession: RN | 0.152 | 0.067 | 2.277 | 0.023 | * |
| Profession: MHP | 0.150 | 0.064 | 2.350 | 0.019 | * |
| Profession: Other | -0.112 | 0.079 | -1.421 | 0.156 |  |
| Age Group: 30-39 | -0.162 | 0.076 | -2.149 | 0.032 | * |
| Age Group: 40-49 | -0.152 | 0.077 | -1.971 | 0.049 | * |
| Age Group: 50-59 | -0.201 | 0.082 | -2.465 | 0.014 | * |
| Age Group: 60-69 | -0.197 | 0.091 | -2.158 | 0.031 | * |
| Age Group: 70+ | -0.350 | 0.134 | -2.606 | 0.009 | ** |
| Previous hallucinogen experience | 0.168 | 0.053 | 3.195 | 0.001 | ** |
| Knowledge check score | 0.256 | 0.024 | 10.432 | <2e-16 | *** |
| Mean concern score | -0.003 | 0.033 | -0.103 | 0.918 |  |

Residual standard error: 0.6233 on 865 degrees of freedom

Multiple R-squared: 0.1842, Adjusted R-squared: 0.172

F-statistic: 15.03 on 13 and 865 DF, p-value: < 2.2e-16

APP = Advance practice provider, RN = registered nurse, MHP = mental health professional.

Significance codes: * = 0.05, ** = 0.01, *** = 0.001

**Table S3. MDMA multivariable linear regression model results.**

| **Coefficient** | **Estimate** | **Standard Error** | **t value** | **Pr(>\|t\|)** | **Significance** |
| --- | --- | --- | --- | --- | --- |
| Intercept | 3.057 | 0.186 | 16.413 | <2e-16 | *** |
| Profession: APP | 0.258 | 0.100 | 2.586 | 0.010 | ** |
| Profession: RN | 0.243 | 0.089 | 2.718 | 0.007 | ** |
| Profession: MHP | 0.273 | 0.085 | 3.223 | 0.001 | ** |
| Profession: Other | -0.009 | 0.105 | -0.090 | 0.928 |  |
| Age Group: 30-39 | -0.200 | 0.100 | -2.008 | 0.045 | * |
| Age Group: 40-49 | -0.163 | 0.102 | -1.602 | 0.109 |  |
| Age Group: 50-59 | -0.250 | 0.108 | -2.321 | 0.021 | * |
| Age Group: 60-69 | -0.147 | 0.121 | -1.216 | 0.224 |  |
| Age Group: 70+ | -0.348 | 0.178 | -1.957 | 0.051 |  |
| Previous hallucinogen experience | 0.216 | 0.069 | 3.115 | 0.002 | ** |
| Knowledge check score | 0.320 | 0.027 | 11.781 | <2e-16 | *** |
| Mean concern score | -0.063 | 0.039 | -1.629 | 0.104 |  |

Residual standard error: 0.8207 on 865 degrees of freedom

Multiple R-squared: 0.2159, Adjusted R-squared: 0.2042

F-statistic: 18.32 on 13 and 865 DF, p-value: < 2.2e-16

APP = Advance practice provider, RN = registered nurse, MHP = mental health professional.

Significance codes: * = 0.05, ** = 0.01, *** = 0.001

**Table S4. Personal and Professional Exposure to Psilocybin and MDMA**

|  | **Psilocybin** | **MDMA** |
| --- | --- | --- |
| **I have patients that use this hallucinogen currently.** |  |  |
| Strongly agree | 69 (7.8%) | 26 (3.0%) |
| Agree | 195 (22.2%) | 134 (15.2%) |
| Neutral | 194 (22.1%) | 177 (20.1%) |
| Disagree | 259 (29.5%) | 274 (31.2%) |
| Strongly disagree | 162 (18.4%) | 268 (30.5%) |
| **I have seen someone under the influence of this hallucinogen:** |  |  |
| In a recreational context | 650 (73.9%) | 522 (59.4%) |
| In a research/clinical setting | 76 (8.6%) | 55 (6.3%) |
| While they were seeking medical care | 68 (7.7%) | 77 (8.8%) |
| Never | 194 (22.1%) | 308 (35.0%) |
| **How would you describe the experience(s) you observed?** |  |  |
| Primarily positive | 529 (77.0%) | 356 (62.0%) |
| Somewhat positive | 88 (12.8%) | 101 (17.6%) |
| Neutral | 47 (6.8%) | 63 (11.0%) |
| Somewhat negative | 20 (2.9%) | 30 (5.2%) |
| Primarily negative | 3 (0.4%) | 24 (4.2%) |

**Figure S1: Attitude and knowledge ratings for psilocybin**

(5=strongly agree, 1=strongly disagree)


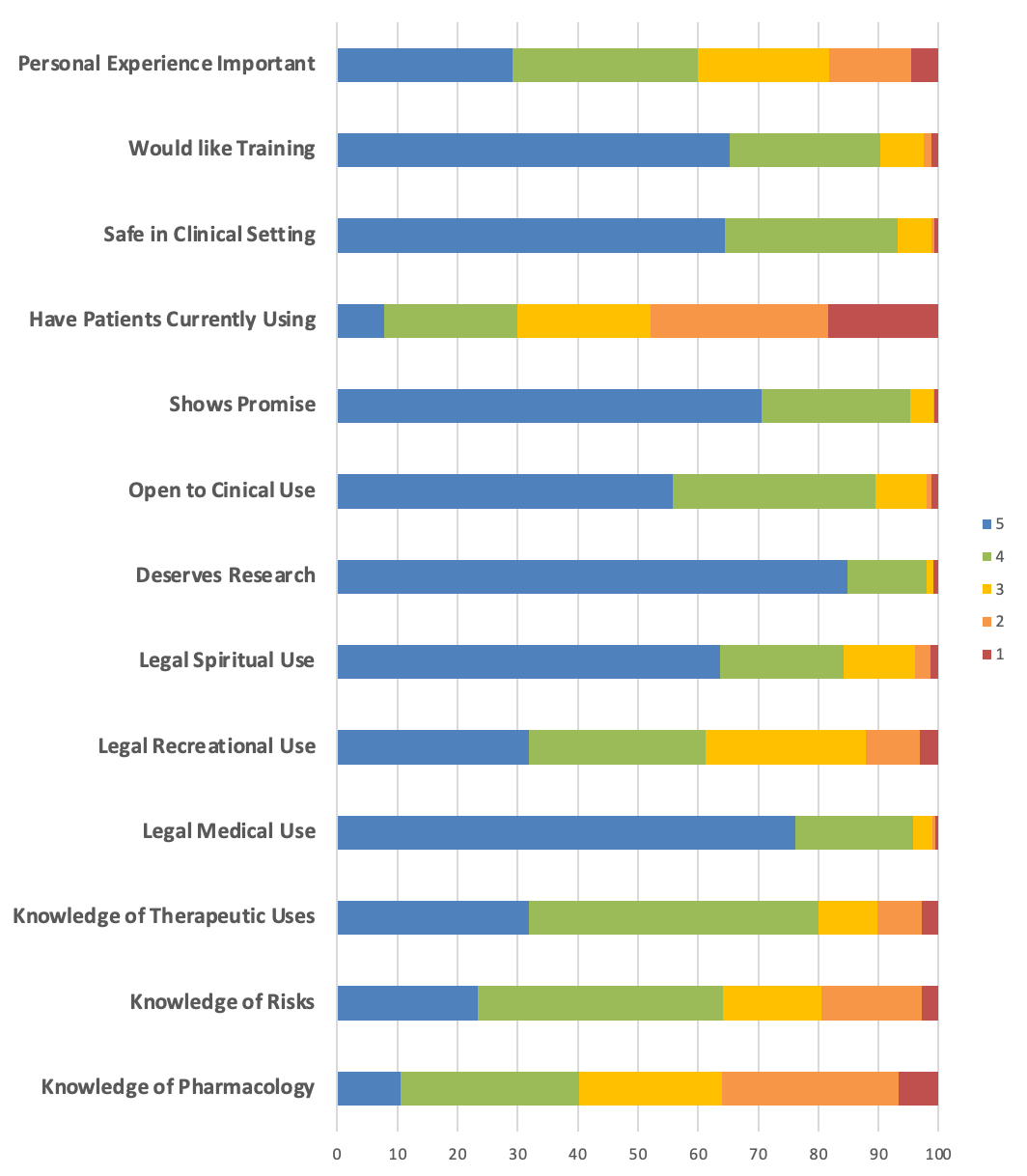


**Figure S2: Attitude and knowledge ratings for MDMA**


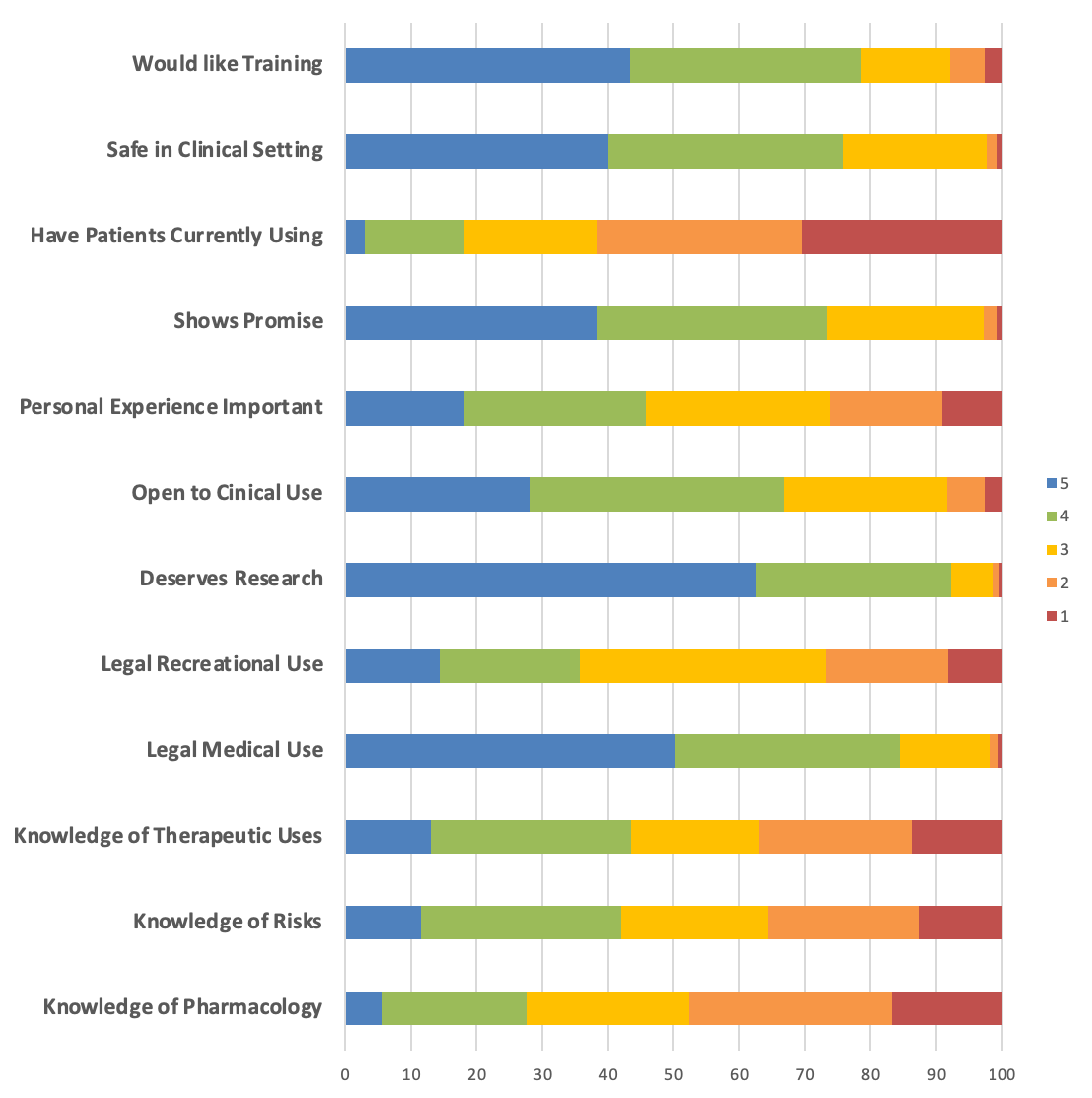


**Figure S3: Attitude and knowledge ratings for psilocybin vs. MDMA**


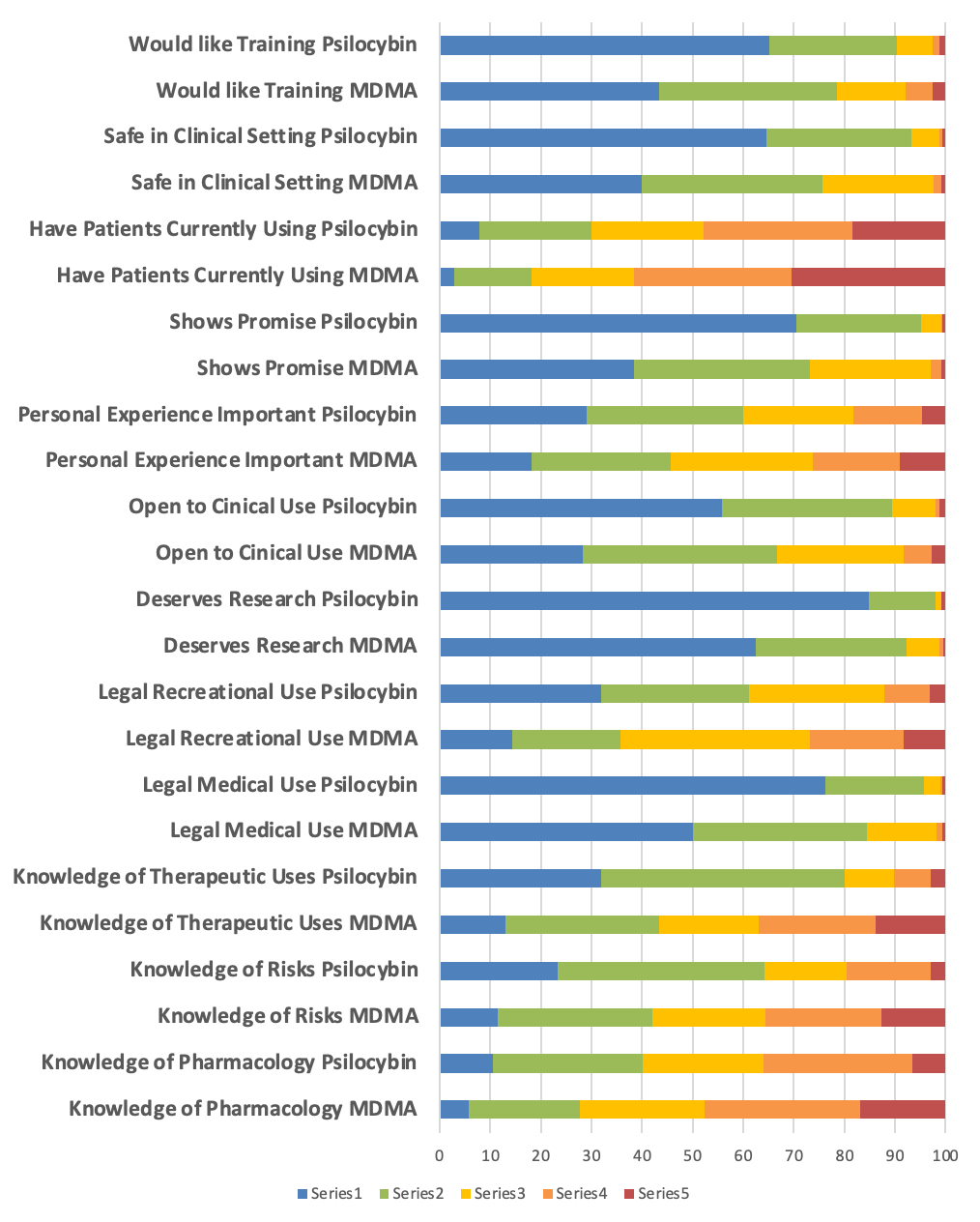


**Figure S4: Knowledge check scores for psilocybin and MDMA (max 3 points)**


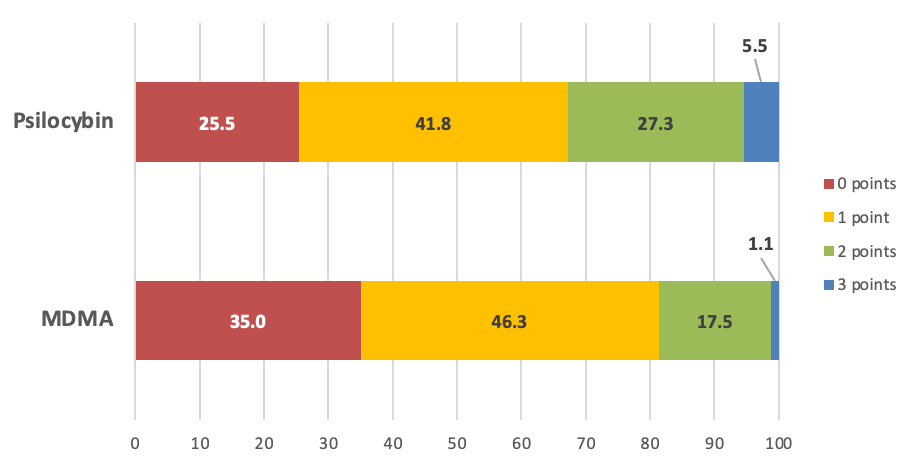

Supplement: Supplementary file 1 — Supplementary Material 1 [file 41598_2024_78736_MOESM1_ESM.docx]
