# Supplementary material for: Knowledge, attitudes, and concerns about psilocybin and MDMA as novel therapies among U.S. healthcare professionals
Source: Sci Rep. 2024 Nov 14;14:28022. doi: 10.1038/s41598-024-78736-1 (PMC11564663; doi:10.1038/s41598-024-78736-1)
Supplement: Supplementary file 2 — Supplementary Material 2 [file 41598_2024_78736_MOESM2_ESM.pdf]

Info/Eligibility

Thank you for your interest in this anonymous online survey study on healthcare professionals' knowledge and attitudes about hallucinogens. This research is being conducted by scientists at Johns Hopkins University School of Medicine and has been deemed "exempt" by the Johns Hopkins University Institutional Review Board (IRB), meaning it involves minimal risk and does not directly involve human subjects because no personally identifiable information (e.g., name, email address, IP address) will be collected in this survey. The greatest risks of this study include boredom or emotional discomfort when answering some questions.

Our Previous Research
Our research team has conducted surveys characterizing positive and challenging experiences after taking psychedelics and other drugs. We've also studied both potential clinical uses and risks of giving psilocybin to volunteers in our laboratory.

Purpose of This Study
The goal of this survey is to learn more about how much healthcare professionals (broadly defined) know about hallucinogens that are being used medically or considered for medical use. We also want to characterize how healthcare professionals view these substances and their potential risks and benefits. For the purposes of this survey, we will be asking specifically about psilocybin (found in "magic mushrooms"), 3,4-methylenedioxymethamphetamine (MDMA), ketamine, and cannabis. For the purposes of this survey, anyone who works in a clinical setting as a healthcare professional or mental health provider is welcome to respond. This includes, but is not limited to physicians, psychologists, counselors, social workers, therapists, nurses, physician's assistants, and emergency medical technicians. The present study is limited to individuals working in the United States.

Inclusion Criteria
You are invited to participate in this survey if you fulfill all of the three criteria listed below.
1) You are at least 18 years old.
2) You read and write English fluently.
3) You work in a clinical setting in the United States as a healthcare professional or mental health provider (i.e., in a role such as physician, psychologist, counselor, social worker,

therapist, nurse, physician's assistant, or emergency medical technician, among others).

What the Study Entails

Participation in this study involves filling out an online survey that will take approximately 20 minutes. You will be required to complete the survey in one sitting.

Why should I participate?

We believe that this study is scientifically important. We would like you to participate because we need to collect responses from many different people. You may find this survey interesting. Although there is no monetary compensation for participation, you will be making a unique and important contribution to science.

Is my participation confidential?

Yes. We do not collect identifying information such as your name, email address, or IP address. Your anonymous responses will be seen and analyzed by Johns Hopkins staff or representatives. The study data will be hosted on a secure website (www.Qualtrics.com). Qualtrics is a reputable and secure platform for conducting survey research online that is approved for use by Johns Hopkins. To further protect the confidentiality of participants, the results of most questions will be combined and presented as group data, rather than individual responses. We may quote from your textual responses. However, if you provide specific identifying information, we will edit your responses to protect your confidentiality.

Benefits

There is no direct benefit to you from being in this study. Responses from many different people and your input could advance our understanding of current knowledge and attitudes surrounding the medical use of hallucinogens. These opinions could be useful to inform future scientific research and policies regarding medical hallucinogen administration.

What kind of information will I be providing?

At the beginning of the survey, you will be asked questions about your background, demographics (age, race, etc.), and career training. We will also ask questions assessing your knowledge and attitudes regarding psilocybin, MDMA, ketamine, and cannabis and their potential risks and benefits in medical care. Finally, we will collect open-ended responses to gather other relevant comments or opinions regarding these substances and their use.

Risks/Discomforts
You may get tired or bored when you are completing the survey. You do not have to answer any question you do not want to answer.

What will become of the results from this study?
The researchers intend to publish the results from this study in the scientific literature and to present results at scientific meetings. We will also make the results publicly available by posting a notice of any scholarly publications on the website of the Johns Hopkins Center for Psychedelic and Consciousness Research (https://HopkinsPsychedelic.org).

Your responses to the survey will only be used if you complete the survey.
Your participation in this study is voluntary. Reviewing this information and completing the survey will serve as your consent to be in the study. Even after you begin the survey, you may stop answering the questions at any time. If you stop early, none of your responses will be used. At the end of the survey, you will have a final opportunity to consent, or not, to all of your responses being submitted. Some attention check items are included in the survey. If you respond incorrectly to any of these, none of your responses will be used.

How do I start?
You can begin the survey by clicking 'Begin survey' at the bottom of this page. This survey will take approximately 20 minutes. You will be required to complete the survey in one sitting. It is important that you complete the survey only once, and that you answer each question honestly and seriously. If you are not ready to complete the survey now, please return to this page at a time that is convenient for you.

You can exit the survey at any time by closing your browser window. If you exit the survey early, your responses will not be used.

During the survey, please do not hit the "back" button on your internet browser as it may erase your answers or prematurely terminate your session.

Contact Information
If you have any questions about this study, please feel free to contact the Principal Investigator Albert Garcia-Romeu, Ph.D. at (410) 550-1972 or agarci33@jhmi.edu.

The IRB can help you if you have questions about your rights as a research participant or if you have other questions, concerns or complaints about this research study. You may contact the IRB at 410-502-2092 or jhmeirb@jhmi.edu.

By clicking 'Begin survey' below, you affirm that:
• you have read the information above,
• you voluntarily agree to participate,
• you are at least 18 years old,
• you read and write English fluently,
• you work in a clinical setting in the United States as a healthcare professional or mental health provider (i.e., in a role such as physician, psychologist, counselor, social worker, therapist, nurse, physician’s assistant, or emergency medical technician, among others).

Please click below to begin or exit the survey.

- ○ Begin survey
- ○ Exit survey

Have you completed this survey before?

- ○ Yes
- ○ No

Do you read, write, and speak English fluently?

- ○ Yes
- ○ No

Are you 18 years old or older?

- ○ Yes
- ○ No

Do you work in a clinical setting in the United States as a healthcare professional or mental health provider (i.e., in a role such as physician, psychologist, counselor, social

worker, therapist, nurse, physician's assistant, or emergency medical technician, among others)?

- ○ Yes
- ○ No

☐ I'm not a robot
reCAPTCHA
Privacy - Terms

For Individuals who do not meet inclusion criteria or opt to exit survey:

We sincerely appreciate your interest in this survey and your willingness to volunteer your time. Unfortunately, you do not currently meet all of the necessary criteria to participate.

In the future, we hope to initiate another version of this survey with less restrictive inclusion requirements. We hope that you may be interested in participating in such a survey in the future.

With gratitude for your interest,
The Johns Hopkins Center for Psychedelic and Consciousness Research

JOHNS HOPKINS
Center for Psychedelic & Consciousness Research

For Individuals who meet inclusion criteria and opt to begin survey:

Demographics

Please answer the following demographics questions.

Age

[▾]

Gender

- Male
- Female
- Transgender male
- Transgender female
- Non-binary
- Other
- Prefer not to answer

What is the highest degree or level of school you have completed?

- Some high school, no diploma
- High school graduate, diploma or the equivalent (GED)
- Some college credit, no degree
- Trade/technical/vocational training
- Associate degree
- Bachelor's degree
- Master's degree
- Professional degree
- Doctorate degree

Which of the following best describes your racial background? (select all that apply)

- White or European American
- Black or African American
- African ancestry
- American Indian or Alaska Native
- Asian Indian
- Chinese
- Filipino
- Japanese
- Korean
- Vietnamese
- Native Hawaiian Guamanian or Chamorro Samoan
- None of these apply to me. I describe my race/ethnicity as:

- ☐ Prefer not to answer

Are you Hispanic or Latino? ('Hispanic' or 'Latino' refers to a person of Cuban, Mexican, Puerto Rican, South or Central American, or other Spanish culture or origin, regardless of race.)

- ○ Yes
- ○ No
- ○ Prefer not to answer

How would you characterize your overall spiritual or religious belief system?

- ○ Agnostic (unsure)
- ○ Atheist (non-believer)
- ○ Buddhism
- ○ Christianity
- ○ Hinduism
- ○ Islam
- ○ Judaism
- ○ Sikhism
- ○ Spiritual but not religious
- ○ ______ Other (please specify)
- ○ Prefer not to answer

What is your total annual household income (in U.S. dollars)?

- ○ $0 to $9,999
- ○ $10,000 to $24,999
- ○ $25,000 to 49,999
- ○ $50,000 to 74,999
- ○ $75,000 to 99,999
- ○ $100,000 to 149,999
- ○ $150,000 and greater
- ○ Prefer not to answer

What is your current professional role?

- Physician
- Psychologist
- Counselor
- Social Worker
- Therapist
- Nurse Practitioner
- Registered Nurse
- Advanced Practice Registered Nurse
- Physician’s Assistant
- Emergency Medical Technician
- ____________ Other

Physician specialty

- Allergy and immunology
- Anesthesiology
- Cardiology
- Dermatology
- Diagnostic radiology
- Emergency medicine
- Family medicine
- Internal medicine
- Medical genetics
- Neurology
- Nuclear medicine
- Obstetrics and gynecology
- Ophthalmology
- Pathology
- Pediatrics
- Physical medicine and rehabilitation
- Preventive medicine
- Psychiatry
- Radiation oncology

- Surgery
- Urology
- [ ] Other
- N/A

In what year did you complete your professional training?

[ ]

In which U.S. region do you currently reside?

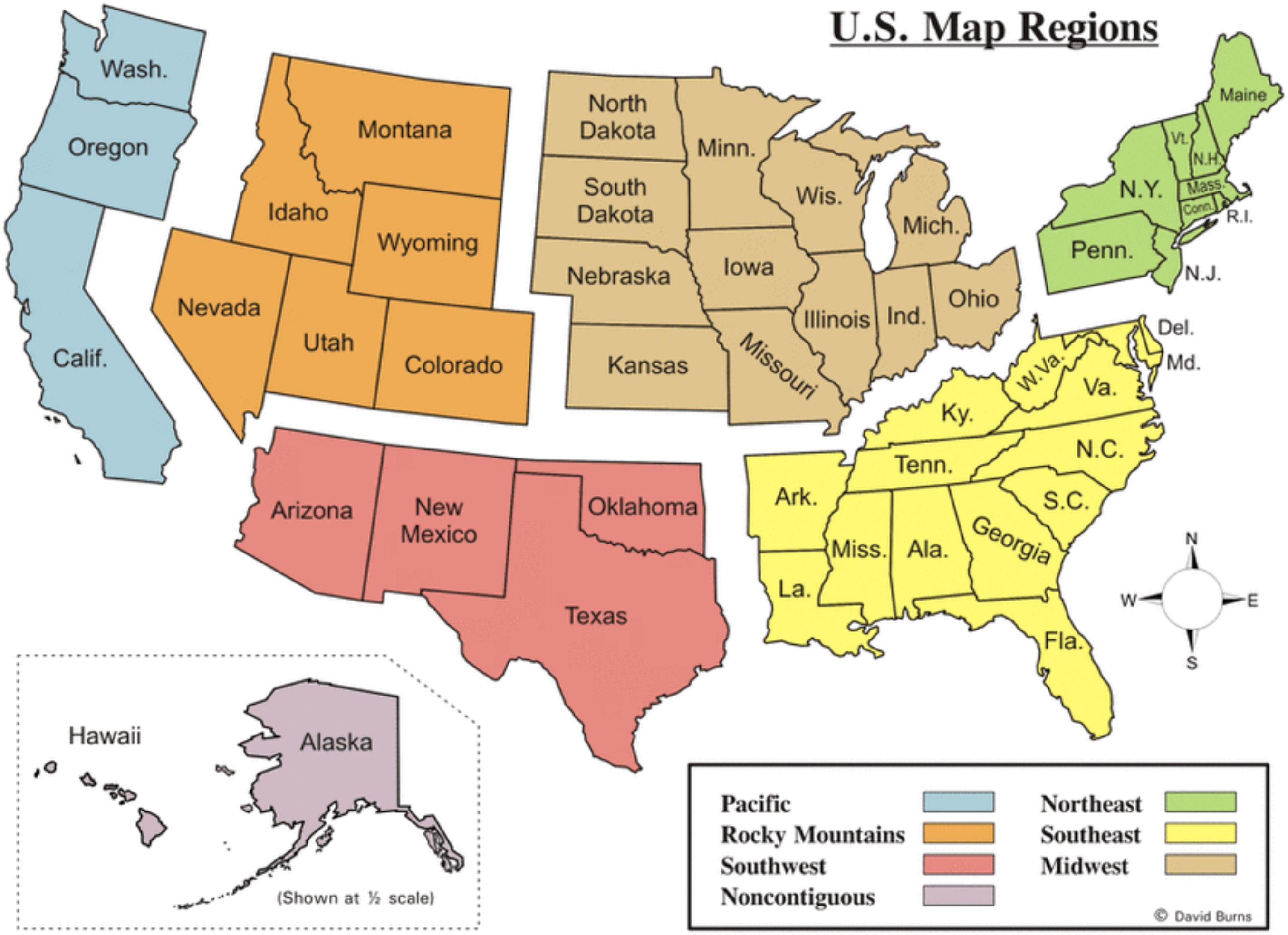

- Pacific
- Rocky Mountains
- Southwest

- Northeast
- Southeast
- Midwest
- Noncontiguous

Do you have prescribing capabilities?

- Yes
- No

Are you currently practicing or seeing patients/clients?

- Yes
- No

Do you conduct research in any capacity?

- Yes
- No

Have you ever taken a hallucinogen?

- Yes
- No
- Prefer not to answer

Psilocybin ("Magic Mushrooms")

These next questions focus on psilocybin (the active ingredient in "magic mushrooms").

Psilocybin

Please rate how strongly you agree with the following statements:

	Strongly disagree	Disagree	Neutral	Agree	Strongly agree
I have good knowledge of the potential therapeutic uses of psilocybin.	O	O	O	O	O
I have good knowledge of the risks and side effects of psilocybin.	O	O	O	O	O
I have good knowledge of the pharmacology of psilocybin.	O	O	O	O	O
I have patients that use psilocybin currently.	O	O	O	O	O
I would be open to using psilocybin as a treatment for my patients.	O	O	O	O	O
I would like to receive training on using psilocybin as a treatment with my patients.	O	O	O	O	O
Psilocybin can be delivered safely in a clinical setting.	O	O	O	O	O
Psilocybin shows promise in treating psychiatric disorders.	O	O	O	O	O
Psilocybin deserves further research for treatment of psychiatric disorders.	O	O	O	O	O
Psilocybin should be legally accessible for spiritual/religious use.	O	O	O	O	O
Psilocybin should be legally accessible for recreational/non-medical use.	O	O	O	O	O
Psilocybin should be legally accessible for supervised medical use.	O	O	O	O	O

	Strongly disagree	Disagree	Neutral	Agree	Strongly agree
Personal experience with psilocybin is important to be able to use it clinically.	O	O	O	O	O

Where has most of your knowledge on psilocybin come from? (select all that apply)

- ☐ Formal clinical training
- ☐ Colleagues
- ☐ Academic literature
- ☐ Conference presentations or workshops
- ☐ Media (e.g., books, films, podcasts, social media, online forums)
- ☐ Informal conversations
- ☐ Past experience with patients
- ☐ Personal experience
- ☐ [ ] Other

What types of sources would you trust for training and information on therapeutic use of psilocybin? (select all that apply)

- ☐ Professional organizations
- ☐ Pharmaceutical companies
- ☐ Academic research centers
- ☐ Experienced clinicians / practitioners
- ☐ Private training institutions
- ☐ [ ] Other

In what settings do you think it would be appropriate to administer psilocybin clinically? (select all that apply)

- ☐ Private practice
- ☐ Hospital (outpatient)
- ☐ Hospital (inpatient)
- ☐ Detox / Drug rehabilitation facilities
- ☐ Specialized clinics

- ☐ Emergency Department
- ☐ At patient's home (unsupervised)
- ☐ At patient's home (supervised)
- ☐ None of these
- ☐ [ ] Other

Please rate the importance of the following potential concerns of administering psilocybin clinically to patients:

	Not at all concerned	Somewhat concerned	Moderately concerned	Very concerned	Extremely concerned
Psychosis	○	○	○	○	○
Addiction	○	○	○	○	○
Stigma	○	○	○	○	○
Financial cost / insurance coverage	○	○	○	○	○
Recreational use / misuse	○	○	○	○	○
	Not at all concerned	Somewhat concerned	Moderately concerned	Very concerned	Extremely concerned
Lack of trained providers	○	○	○	○	○
Time required to administer	○	○	○	○	○
Administration to patients with contraindications	○	○	○	○	○
Exploitation of patients	○	○	○	○	○
Other [ ]	○	○	○	○	○

Have you ever seen someone under the influence of psilocybin? (select all that apply)

- ☐ No, never
- ☐ Yes, while they were seeking medical care
- ☐ Yes, in a recreational context
- ☐ Yes, in a research / clinical setting

How would you describe the experience(s) you observed?

- ○ Primarily positive
- ○ Somewhat positive
- ○ Neutral
- ○ Somewhat negative
- ○ Primarily negative

Knowledge Check

Current data indicate psilocybin may be useful for treating (select all that apply):

- ☐ Schizophrenia spectrum and other psychotic disorders
- ☐ Bipolar and related disorders
- ☐ Depressive disorders
- ☐ Anxiety disorders
- ☐ Somatic symptoms and related disorders
- ☐ Substance-related and addictive disorders
- ☐ Personality disorders
- ☐ Management of acute agitation
- ☐ Palliative care

Risks of psilocybin may include (select all that apply):

- ☐ Increased blood pressure and heart rate
- ☐ Headache
- ☐ Transient fear or anxiety
- ☐ Weight gain
- ☐ Rash
- ☐ Nosebleed
- ☐ Constipation

The primary mechanism by which psilocybin is thought to work is (select all that apply):

- ☐ Activity at serotonin receptors
- ☐ Activity at dopamine receptors
- ☐ Activity at CB1 and CB2 receptors

- ☐ Activity at NMDA receptors
- ☐ Activity at opioid receptors

Which of the following is a common house pet?

- ○ Turkey
- ○ Pig
- ○ Cat
- ○ Monkey
- ○ Zebra

Would you like to elaborate or provide any additional thoughts on the therapeutic use of psilocybin?

- ○ Yes ______
- ○ No

Information & Resources on Psilocybin

Thanks for completing this section! Psilocybin is a naturally occurring serotonin 2A receptor agonist that has shown promise for treating anxiety in people with serious illness (Griffiths et al., 2016; Grob et al., 2011; Ross et al., 2016), depression (Carhart-Harris et al., 2021; Davis et al., 2021), and substance use disorders such as alcohol and tobacco dependence (Bogenschutz et al., 2015; Johnson et al, 2014). It is primarily administered orally in doses of around 20 - 30mg under supervised, psychologically supportive conditions, with drug effects lasting approximately 6 hours (Garcia-Romeu & Richards, 2018). Psilocybin is not addictive and largely considered physiologically non-toxic (Passie, 2002). However, psilocybin can interact with serotonergic medications and is thought to be contraindicated for individuals with cardiovascular conditions, or personal or family history of psychotic illness or bipolar mood (Johnson et al., 2018). Onto the next section!

References

Bogenschutz, M. P., Forcehimes, A. A., Pommy, J. A., Wilcox, C. E., Barbosa, P. C. R., & Strassman, R. J. (2015). Psilocybin-assisted treatment for alcohol dependence: a proof-of-concept study. *Journal of psychopharmacology*, *29*(3), 289-299. https://journals.sagepub.com/doi/full/10.1177/0269881114565144

Carhart-Harris, R., Giribaldi, B., Watts, R., Baker-Jones, M., Murphy-Beiner, A., Murphy, R., ... & Nutt, D. J. (2021). Trial of psilocybin versus escitalopram for depression. New England Journal of Medicine, 384(15), 1402-1411. https://www.nejm.org/doi/full/10.1056/NEJMoa2032994

Davis, A. K., Barrett, F. S., May, D. G., Cosimano, M. P., Sepeda, N. D., Johnson, M. W., ... & Griffiths, R. R. (2021). Effects of psilocybin-assisted therapy on major depressive disorder: a randomized clinical trial. JAMA psychiatry, 78(5), 481-489. https://jamanetwork.com/journals/jamapsychiatry/article-abstract/2772630

Garcia-Romeu, A., & Richards, W. A. (2018). Current perspectives on psychedelic therapy: use of serotonergic hallucinogens in clinical interventions. International Review of Psychiatry, 30(4), 291-316. https://www.tandfonline.com/doi/full/10.1080/09540261.2018.1486289

Griffiths, R. R., Johnson, M. W., Carducci, M. A., Umbricht, A., Richards, W. A., Richards, B. D., ... & Klinedinst, M. A. (2016). Psilocybin produces substantial and sustained decreases in depression and anxiety in patients with life-threatening cancer: A randomized double-blind trial. Journal of psychopharmacology, 30(12), 1181-1197. https://journals.sagepub.com/doi/full/10.1177/0269881116675513

Grob, C. S., Danforth, A. L., Chopra, G. S., Hagerty, M., McKay, C. R., Halberstadt, A. L., & Greer, G. R. (2011). Pilot study of psilocybin treatment for anxiety in patients with advanced-stage cancer. Archives of general psychiatry, 68(1), 71-78. https://jamanetwork.com/journals/jamapsychiatry/article-abstract/210962

Johnson, M. W., Garcia-Romeu, A., Cosimano, M. P., & Griffiths, R. R. (2014). Pilot study of the 5-HT2AR agonist psilocybin in the treatment of tobacco addiction. Journal of psychopharmacology, 28(11), 983-992. https://journals.sagepub.com/doi/full/10.1177/0269881114548296

Johnson, M. W., Griffiths, R. R., Hendricks, P. S., & Henningfield, J. E. (2018). The abuse potential of medical psilocybin according to the 8 factors of the Controlled Substances Act. Neuropharmacology, 142, 143-166. https://www.sciencedirect.com/science/article/pii/S0028390818302296

Passie, T., Seifert, J., Schneider, U., & Emrich, H. M. (2002). The pharmacology of psilocybin. *Addiction biology*, *7*(4), 357-364. https://onlinelibrary.wiley.com/doi/abs/10.1080/1355621021000005937

Ross, S., Bossis, A., Guss, J., Agin-Liebes, G., Malone, T., Cohen, B., ... & Schmidt, B. L. (2016). Rapid and sustained symptom reduction following psilocybin treatment for anxiety and depression in patients with life-threatening cancer: a randomized controlled trial. Journal of psychopharmacology, 30(12), 1165-1180. https://journals.sagepub.com/doi/full/10.1177/0269881116675512

MDMA ("Ecstasy" or "Molly")

These next questions focus on MDMA ("ecstasy" or "molly").

MDMA

Please rate how strongly you agree with the following statements:

	Strongly disagree	Disagree	Neutral	Agree	Strongly agree
I have good knowledge of the potential therapeutic uses of MDMA.	O	O	O	O	O
I have good knowledge of the risks and side effects of MDMA.	O	O	O	O	O
I have good knowledge of the pharmacology of MDMA.	O	O	O	O	O
I have patients that use MDMA currently.	O	O	O	O	O
	Strongly disagree	Disagree	Neutral	Agree	Strongly agree

	Strongly disagree	Disagree	Neutral	Agree	Strongly agree
I would be open to using MDMA as a treatment for my patients.	O	O	O	O	O
I would like to receive training on using MDMA as a treatment with my patients.	O	O	O	O	O
MDMA can be delivered safely in a clinical setting.	O	O	O	O	O
MDMA shows promise in treating psychiatric disorders.	O	O	O	O	O

	Strongly disagree	Disagree	Neutral	Agree	Strongly agree
MDMA deserves further research for treatment of psychiatric disorders.	O	O	O	O	O
MDMA should be legally accessible for recreational/non-medical use.	O	O	O	O	O
MDMA should be legally accessible for supervised medical use.	O	O	O	O	O
Personal experience with MDMA is important to be able to use it clinically.	O	O	O	O	O

Where has most of your knowledge on MDMA come from? (select all that apply)

- ☐ Formal clinical training
- ☐ Colleagues
- ☐ Academic literature
- ☐ Conference presentations or workshops
- ☐ Media (e.g., books, films, podcasts, social media, online forums)
- ☐ Informal conversations
- ☐ Past experience with patients

- ☐ Personal experience
- ☐ [   ] Other

What types of sources would you trust for training and information on therapeutic use of MDMA? (select all that apply)

- ☐ Professional organizations
- ☐ Pharmaceutical companies
- ☐ Academic research centers
- ☐ Experienced clinicians / practitioners
- ☐ Private training institutions
- ☐ [   ] Other

In what settings do you think it would be appropriate to administer MDMA clinically? (select all that apply)

- ☐ Private practice
- ☐ Hospital (outpatient)
- ☐ Hospital (inpatient)
- ☐ Detox / Drug rehabilitation facilities
- ☐ Specialized clinics
- ☐ Emergency Department
- ☐ At patient’s home (unsupervised)
- ☐ At patient’s home (supervised)
- ☐ None of these
- ☐ [   ] Other

Please rate the importance of the following potential concerns of administering MDMA clinically to patients:

	Not at all concerned	Somewhat concerned	Moderately concerned	Very concerned	Extremely concerned
Psychosis	○	○	○	○	○
Addiction	○	○	○	○	○
Stigma	○	○	○	○	○

	Not at all concerned	Somewhat concerned	Moderately concerned	Very concerned	Extremely concerned
Financial cost / insurance coverage	○	○	○	○	○
Recreational use / misuse	○	○	○	○	○
	Not at all concerned	Somewhat concerned	Moderately concerned	Very concerned	Extremely concerned
Lack of trained providers	○	○	○	○	○
Time required to administer	○	○	○	○	○
Administration to patients with contraindications	○	○	○	○	○
Exploitation of patients	○	○	○	○	○
Other ______	○	○	○	○	○

Have you ever seen someone under the influence of MDMA? (select all that apply)

- ☐ No, never
- ☐ Yes, while they were seeking medical care
- ☐ Yes, in a recreational context
- ☐ Yes, in a research / clinical setting

How would you describe the experience(s) you observed?

- ○ Primarily positive
- ○ Somewhat positive
- ○ Neutral
- ○ Somewhat negative
- ○ Primarily negative

Knowledge Check

Current data indicate MDMA may be useful for treating (select all that apply):

- ☐ Schizophrenia spectrum and other psychotic disorders

- ☐ Bipolar and related disorders
- ☐ Depressive disorders
- ☐ Post-traumatic stress disorder (PTSD)
- ☐ Somatic symptoms and related disorders
- ☐ Substance-related and addictive disorders
- ☐ Personality disorders
- ☐ Management of acute agitation
- ☐ Social anxiety in autism spectrum disorder

Risks of MDMA may include (select all that apply):

- ☐ Increased blood pressure and heart rate
- ☐ Headache
- ☐ Hyperthermia
- ☐ Weight gain
- ☐ Rash
- ☐ Nosebleed
- ☐ Constipation

The primary mechanism by which MDMA is thought to work is (select all that apply):

- ☐ Activity at monoamine neurotransmitter sites
- ☐ Activity at CB1 and CB2 receptors
- ☐ Activity at NMDA receptors
- ☐ Activity at opioid receptors

Would you like to elaborate or provide any additional thoughts on the therapeutic use of MDMA?

- ○ Yes
- ○ No

Information & Resources on MDMA

Thank you for completing this section! MDMA is a synthetic amphetamine that, unlike other amphetamines, has unique effects on the serotonin system. The drug's subjective effects include increased sociability, energy, and feelings of empathy (de la Torre et al., 2004; Dumont et al., 2009; Kamilar & Bedi, 2015). Although first synthesized in 1912, it became well known as an adjunct to psychotherapy in the 1960s, before expanding recreational use led to it being added to Schedule I in 1985. It has lately shown great promise in treating PTSD, including a recently completed phase III trial (Mitchell et al., 2021). More preliminarily, it has shown some promise in the treatment of social anxiety in autism (Danforth et al., 2018), and alcohol use disorder (Sessa et al., 2019).

MDMA is typically administered orally in a dose of 75-125 mg under supervised, psychologically supportive conditions, with drug effects lasting up to 8 hours (Mitchell et al., 202; Sessa et al., 2021; Danforth 2018). Recent exposure to antidepressant drugs that target reuptake transporters may reduce treatment response to MDMA-assisted psychotherapy (Feduccia et al., 2021).

References

Danforth, A. L., Grob, C. S., Struble, C., Feduccia, A. A., Walker, N., Jerome, L., ... & Emerson, A. (2018). Reduction in social anxiety after MDMA-assisted psychotherapy with autistic adults: a randomized, double-blind, placebo-controlled pilot study. Psychopharmacology, 235(11), 3137-3148. https://link.springer.com/article/10.1007/s00213-018-5010-9

de la Torre R, Farré M, Roset PN, Pizarro N, Abanades S, Segura M, Segura J, Camí J. Human pharmacology of MDMA: pharmacokinetics, metabolism, and disposition. Ther Drug Monit. 2004 Apr;26(2):137-44. doi: 10.1097/00007691-200404000-00009. PMID: 15228154. https://pubmed.ncbi.nlm.nih.gov/15228154/

Dumont GJ, Sweep FC, van der Steen R, Hermsen R, Donders AR, Touw DJ, van Gerven JM, Buitelaar JK, Verkes RJ. Increased oxytocin concentrations and prosocial feelings in humans after ecstasy (3,4-methylenedioxymethamphetamine) administration. Soc Neurosci. 2009;4(4):359-66. doi: 10.1080/17470910802649470. PMID: 19562632. https://pubmed.ncbi.nlm.nih.gov/19562632/

Feduccia, A. A., Jerome, L., Mithoefer, M. C., & Holland, J. (2021). Discontinuation of medications classified as reuptake inhibitors affects treatment response of MDMA-assisted psychotherapy. Psychopharmacology, 238(2), 581-588. https://pubmed.ncbi.nlm.nih.gov/33221932/

Kamilar-Britt P, Bedi G. The prosocial effects of 3,4-methylenedioxymethamphetamine (MDMA): Controlled studies in humans and laboratory animals. Neurosci Biobehav Rev. 2015 Oct;57:433-46. doi: 10.1016/j.neubiorev.2015.08.016. Epub 2015 Sep 25. PMID: 26408071; PMCID: PMC4678620. https://pubmed.ncbi.nlm.nih.gov/26408071/

Mitchell, J. M., Bogenschutz, M., Lilienstein, A., Harrison, C., Kleiman, S., Parker-Guilbert, K., ... & Doblin, R. (2021). MDMA-assisted therapy for severe PTSD: a randomized, double-blind, placebo-controlled phase 3 study. Nature Medicine, 27(6), 1025-1033. https://www.nature.com/articles/s41591-021-01336-3

Sessa, B., Higbed, L., O’Brien, S., Durant, C., Sakal, C., Titheradge, D., ... & Nutt, D. J. (2021). First study of safety and tolerability of 3, 4-methylenedioxymethamphetamine-assisted psychotherapy in patients with alcohol use disorder. Journal of Psychopharmacology, 35(4), 375-383. https://pubmed.ncbi.nlm.nih.gov/33601929/

Quality Check

Did you have any issues completing the survey that would make your responses inaccurate or invalid?

- [ ] ____________ Yes (please explain)
- No

Did you have any trouble understanding the questions that would make your responses inaccurate or invalid?

- ____________ Yes (please explain)
- No

Did you answer the questions honestly and accurately to the best of your knowledge?

- Yes
- No

For Individuals who indicate their responses are inaccurate or invalid:

We sincerely appreciate your interest in this survey and your willingness to volunteer your time. Unfortunately, you do not currently meet all of the necessary criteria to participate.

Because you indicated issues with your survey responses, these will not be included in the final analysis.

With gratitude for your interest,
The Johns Hopkins Center for Psychedelic and Consciousness Research

JOHNS HOPKINS
Center for Psychedelic & Consciousness Research

For Individuals who complete the survey and indicate their responses are valid:

You have completed the survey! Thanks for your response!

We sincerely appreciate your time and effort completing the survey and look forward to publishing the results.

With gratitude,
The Johns Hopkins Center for Psychedelic and Consciousness Research

JOHNS HOPKINS
Center for Psychedelic & Consciousness Research

Powered by Qualtrics
